# Challenges in the Immune System: Mesoscale and Mesoregime Complexity

**DOI:** 10.1002/cai2.70030

**Published:** 2025-10-20

**Authors:** Ying Ren, Ai‐Guo Wu, Yu Shi, Yi‐Fang Ping, Jing‐Hai Li, Xiu‐Wu Bian

**Affiliations:** ^1^ State Key Laboratory of Mesoscience and Engineering, Institute of Process Engineering, Chinese Academy of Sciences Beijing China; ^2^ School of Chemical Engineering, University of Chinese Academy of Sciences Beijing China; ^3^ Ningbo Key Laboratory of Biomedical Imaging Probe Materials and Technology, Ningbo Institute of Materials Technology and Engineering, Chinese Academy of Sciences Ningbo Zhejiang China; ^4^ Institute of Pathology, Southwest Hospital Third Military Medical University Chongqing China

**Keywords:** immune system, mesoregime, mesoscale, mesoscience, research paradigm

## Abstract

One of the greatest challenges in bioscience is to gain a unified view of the human immune system. This paper presents a perspective on the multilevel and multiscale complexities inherent in the immune system and discusses how these features influence the traditional research methodologies rooted in reductionism and holism. It is acknowledged that diverse complexities of the immune system are multilevel in nature, with each level showing multiscale properties, and the complexity emerging always at mesoscales in mesoregimes. Mesoscience encompasses both mesoscales, which represent the intermediate scales between the element scales and system scales at different levels, and mesoregimes, defined as transitional regimes governed by the interplay of at least two dominant mechanisms between two limiting regimes at each level. Therefore, mesoscience provides a promising paradigm to study the complexity and diversity of the immune system by bridging the gaps across multiple hierarchical levels. In particular, we focus on the mesoscience methodology to address the complexities in the immune system and offer insights into potential diagnostic, therapeutic, and theranostic strategies for immune‐related diseases from a mesoscience perspective.

## Graphical abstract

The immune system is multilevel in nature, with each level showing multiscale properties, and the complexity emerging always at mesoscales in mesoregimes governed by the compromise in competition between at least two dominant mechanisms at each level.

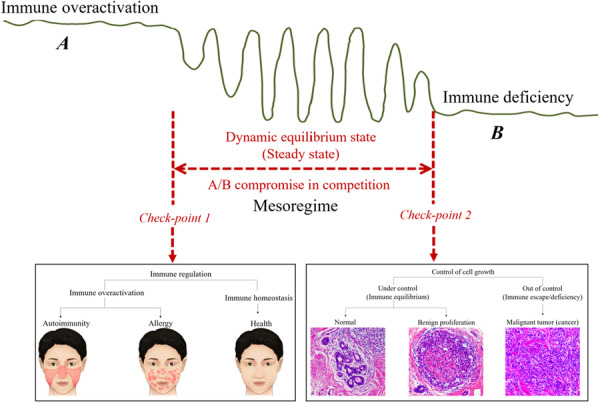

## 1 The Concept of Mesoscience

The complex physical world, including human beings themselves, shows multilevel and multiscale spatiotemporal dynamic structures. Establishing the relationships between multiple scales at each level and the connections between different levels is a major challenge to the understanding, control, and manipulation of complex systems in modern science.

The prefix “meso” of the word “Mesoscience” is derived from the ancient Greek adjective μέσoν (*méson*) or noun μέσo (*méso*), meaning middle or intermediate, and has diverse uses in meso‐containing terms in various fields that can be classified into two categories: one in which meso‐ is only a superficial label, implying the middle (e.g., position, size, state, and extent), and the other in which meso‐ is relevant to the complexity and diversity caused by collective effects, that is, the exact mesoscale problems of importance with respect to mesoscience [1].

**Figure 1:**
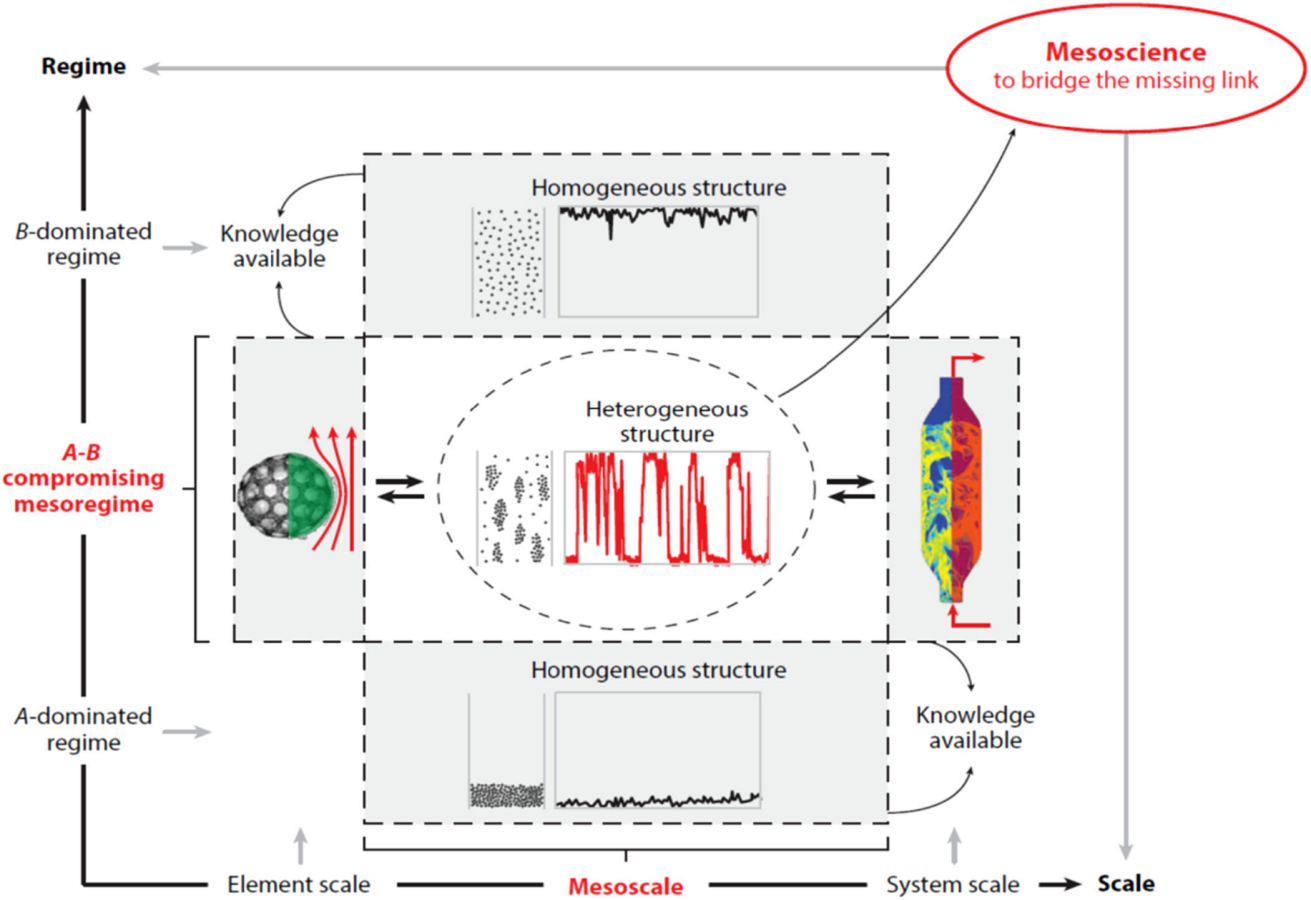
Schematic diagram of the scope of mesoscience in chemical engineering [1].

Based on long‐term exploration beginning in the 1980s, mesoscience has been proposed as an alternative paradigm to tackle critical challenges, with the aim of exploring common principles at level‐specific mesoscales and mesoregimes [2, 3, 4, 5]. Figure 1 shows the scope of mesoscience, exemplified by the identification of the mesoscale of mesoregimes at the chemical reactor level in chemical engineering. Mesoscience refers not only to mesoscales between the scales of elements and systems at different levels but also to mesoregimes, which are governed jointly by at least two dominant mechanisms and lie between two limiting regimes at each level, where only one mechanism dominates. Mesoscale does not refer to an absolute physical scale but is a relative one, referring to the scale ranging between the micro (element) scale and the macro (system) scale. At the mesoscale of the corresponding level, there exists a characteristic structure, namely, the mesoscale structure, which has spatiotemporal dynamic heterogeneity and is crucial to the performance of the structure and thus the properties of the macro (system) scale. Mesoregime refers to the intermediate regime between the other two regimes exclusively dominated by A or B, respectively. For either A‐ or B‐dominated regimes, theory is available with a single variational criterion. However, the A–B compromising regime (mesoregime) featuring at least two dominant mechanisms needs a new theory, that is, mesoscience [6, 7].

It has been recognized that the mesoscale complexity cannot be tackled using an average‐based holism approach due to its blurring of the inherent interactions at different scales into a single one that does not reflect the diversity of system behaviors; moreover, reductionism approaches purely based on the details at the element scale are unable to reach the scalability required for the whole system [8]. Therefore, the goal of mesoscience is to develop a universal principle for the complex physical world, building a bridge for diverse disciplines at different levels.

The breakthrough in understanding complex systems can only be achieved through trans‐level and ‐scale studies at all scales, with a special focus on mesoscale structures and mesoregime characteristics at each hierarchical level. With the aforementioned considerations, there shall be no exception for the immune system.

## 2 The Immune System and Immunity

Immunity, if defined broadly, encompasses all mechanisms and responses used by the body to defend itself against foreign substances, microorganisms, toxins, and noncompatible living cells. The generalized primary forms of host defense are termed “innate,” “inborn,” or “nonspecific” immunity, which guards the body by contributing protective responses that are effective against a diverse variety of threats [9]. The second, or subsequent, form of host defense is termed “adaptive,” “acquired,” or “antigen‐specific” immunity, which is delivered by the immune system itself, with its complex and highly interactive network of lymphocyte species and their products [10].

The immune system is not limited to a single set of specialized cells with discrete functions, but can be considered to be represented by the whole body—each cell in the body has its own internal immune response [11]. It resists not only external threats but also internal challenges. Apart from fighting viruses, bacteria, fungi, and parasites, the immune system also plays other roles such as tissue repair, wound healing, elimination of dead and cancer cells, and formation of healthy gut microbiota. Furthermore, over the lifetime of an individual, these immune mechanisms change, first to adapt to the change from a fetus to an infant, and then to mature and expand during growth, subtly changing in pregnancy, and finally decreasing in senescence [12]. Correspondingly, the immune system can be regarded as a system that maintains homeostasis within the body.

To maintain homeostasis, the immune system operates as a dynamic and multilevel biological system [13, 14], spanning biomolecular, cellular, organ, and organismal levels. Each level is multiscaled with corresponding mesoscale structures, for example, biological structures with spatiotemporally evolving boundaries at different levels [15]. In addition, each level constitutes a subsystem consisting of the element scale and the system scale, and the mesoscale in between, which is a common feature of all complex systems according to mesoscience. As previously discussed [5], the mesoscale of these levels refers to local molecular structures, organelles, tissues, and functional subsystems.

To cope with the challenge of the complexity of the immune system, an understanding of mesoscale interactions and identification of mesoregimes at each level are of crucial importance. From this point of view, some possible links between mesoscience studies and the immune system, together with the consequent challenges in immunological study, are elucidated below.

## 3 Challenges in Immunological Study

Here, we identify mesoscale complexities in mesoregimes across multiple characteristic levels in the immune system, and then point out some possible problems that hamper further progress in immune research and reveal the underlying common traits that may induce complexities in these fields. From a mesoscience perspective, we believe that the current research paradigm cannot fulfill the requirement of the complexity of immune research, and mesoscience might provide a promising paradigm to tackle multiscale spatiotemporal structures and dynamics of the immune system.

### 3.1 Mesoscale Gaps in Immunological Research

The immune system can be systematically analyzed across at least four levels, namely, biomolecular, cellular, organ, and organismal levels. At the biomolecular level, the mesoscale represents local structural domains (e.g., antigen‐binding sites, signalosomes) that govern the spatiotemporal organization critical for the immunological functionality of macromolecules like antibodies, TCRs, or cytokine–receptor complexes. At the cellular level, the mesoscale involves specialized subcellular compartments (e.g., immunological synapses, inflammasomes) that enable regulation of immune cell activation, signaling, and effector functions. At the organ level, the mesoscale refers to tissues of organized immune cells and intercellular materials, where immune cells and stromal components are architecturally organized to mediate antigen presentation, lymphocyte differentiation, and immune surveillance. At the organism level, mesoscale encompasses the dynamic interorgan communication between primary and secondary lymphoid organs (e.g., thymus, spleen, and lymph nodes), forming functionally specialized immune networks that interface with the respiratory, gastrointestinal, cardiovascular, and neuroendocrine systems.

Though the immune system is multilevel and multiscale in nature, current immunological research remains largely focused on biomolecular and cellular levels, and more attention should be paid to the mesoscale problems at other levels.

Currently, considerable omics data are being generated and analyzed at macromolecular and cellular levels, resulting in promising development of genomes, transcriptomes, proteomes, and so forth, which, for the most part, are assessed individually using distinct approaches, generating monothematic rather than integrated knowledge [16]. Biomolecular studies focus on interactions between the various macromolecular systems of a cell, including the interrelationships of DNA, RNA, and protein synthesis and the regulations of these interactions, such as the significance of introns and exons or coding issues during transcription and translation. Cellular studies include investigations of innate and acquired (adaptive) immunity, the cellular communication pathways involved in immunity, cellular recognition, and interactions between antigens and antibodies. However, there are few quantitative studies on other levels, and even fewer investigations on the coupling mechanism across different levels.

For example, as one of the most important scientific problems in immunoregulation, the intake of nutrients and drugs (including Traditional Chinese Medicine, TCM) can improve the microenvironment of different levels and thus affect human health [17, 18]. At the macromolecular level, many investigations are dedicated to the discovery of the molecular mechanisms of nutrients and drug molecules, such as the target site for these molecules, binding constant, signal transduction, drug metabolism, and so forth [19]. At the cellular level, the effects of vitamins, minerals, and drugs on some specific cells can also be assessed [20, 21], and it has been concluded that vitamin D is involved in the development of regulatory T cells, while the vitamin A pathway is involved in signals driving mucosal defense [22]. Nevertheless, few studies pay attention to the effect of nutrients and drugs on different types of cells located within different types of tissues and various derivative effects correspondingly.

Even under normal physiological conditions, effective bio‐distribution and drug delivery are difficult to achieve, as the drug particles face physical and biological barriers—including shear forces, protein adsorption, and rapid clearance—that limit the fraction of administered nanoparticles that reach the target therapeutic sites [23]. These barriers, or boundaries, are often related to the extent and nature of immune cells in the diseased environment present and are thus difficult to overcome with a generalized, one‐size‐fits‐all approach [15, 24, 25].

Therefore, a thorough and complete understanding of the immune system must be based on in‐depth investigations of these subsystems at different levels. For each level, traditional averaging methods are inadequate. Instead, mesoscale structural changes at each level and the corresponding dominant mechanisms should be studied [4, 5]. A combination of advanced experimental and computational methods can be used to study the mesoscopic structures across different levels of the immune system. For example, high‐resolution intravital imaging can be used to track the dynamic structural and functional changes of immune cell clusters, spatial proteomics can elucidate the molecular concentration gradient distribution within the tissue microenvironment, and the data obtained from these experiments can serve as input for corresponding numerical calculations. Among various computational methods, molecular dynamics simulations serve as a computational microscope to detect structural changes of molecular assemblies, multiscale coupling algorithms to correlate local structures with system functions, and agent‐based models to simulate cell interactions by treating individual cells as autonomous agents with intercellular interactions and specific behavioral rules, while multiscale calculations with considerations of compromise between different mechanisms may couple the spatiotemporal dynamics arising from the mesoscale structures at each level.

To achieve a comprehensive understanding of the immune system through trans‐level and ‐scale studies at all scales, the following questions should be answered: How does the elements' behavior relate to the subsystem behavior at each level? What is the interaction between the element and system at each level? How does the mesoscale behavior affect the functionality of the subsystem and then the entire immune system? How can the subsystems be correlated at different levels? This is the common challenge widely present in many complex problems [1, 26], with the immune system showing all characteristic features of such multiscale problems.

### 3.2 Demarcation and Boundaries in the Immune System

Rational demarcation of the levels and scales within the immune system, along with precise identification of the input/output relationships and boundary conditions at each subsystem across all levels, is essential for a comprehensive understanding of immune system complexities. Clearly defining and delineating boundaries within the immune system are critical to elucidate both the intension and extension of the system and its key factors. For instance, in autoimmune diseases, the immune system erroneously attacks self‐tissue boundaries, whereas in infectious diseases, the body's boundary defense function is impaired, leading to an inability to effectively resist external pathogens' invasion. This subsequently results in tissue damage and functional impairment. These disease states underscore the pivotal role of boundary functions in maintaining homeostasis and protecting against external threats, as well as the profound impact of boundary dysfunction on immune‐related pathological processes.

**Figure 2:**
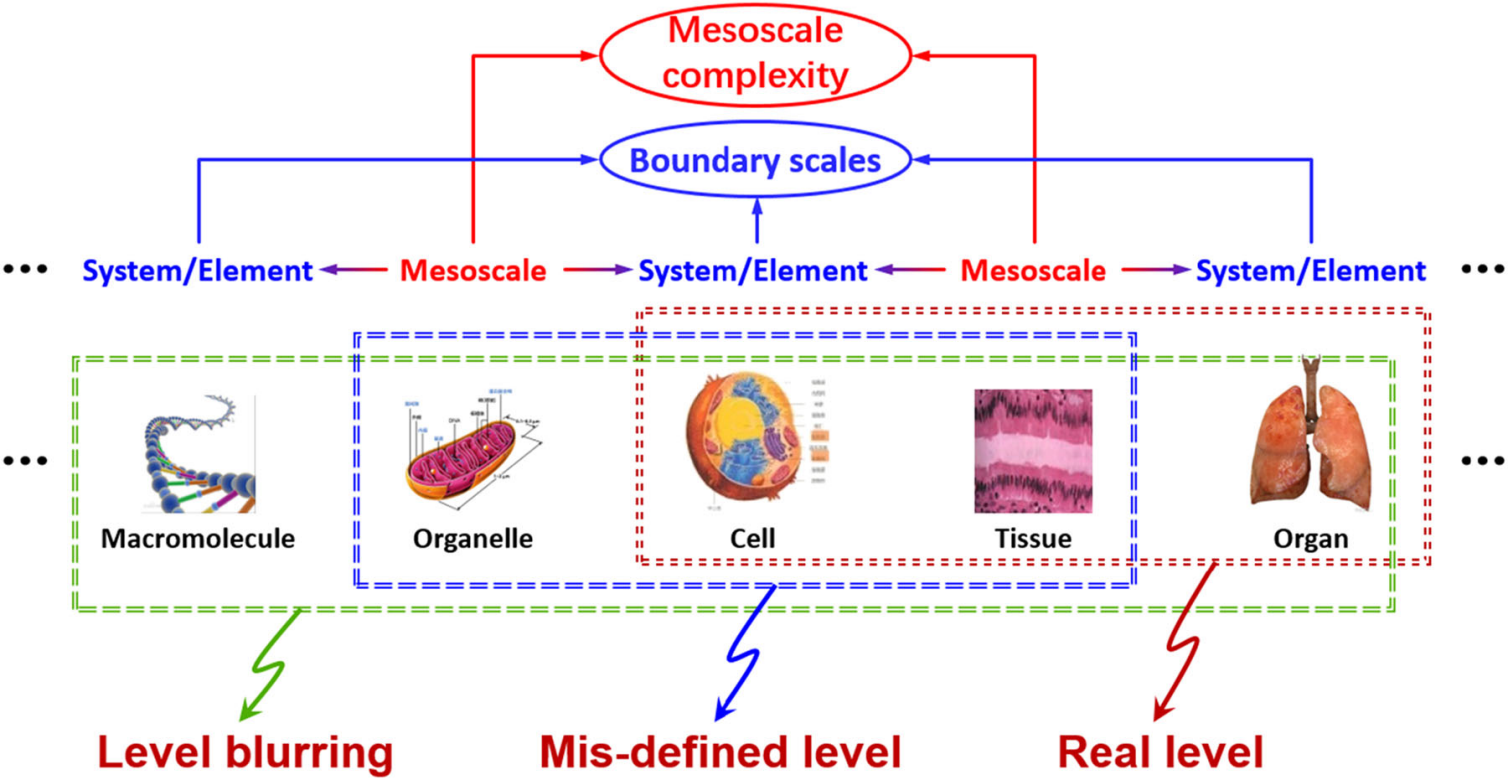
Misdefinition of levels in the immune system (only two levels are listed for reference) [5].

Currently, the definition of biological levels and scales in immunological research remains somewhat arbitrary, often conflating different levels or improperly combining multiple scales into a single level, as shown in Figure 2. For example, the “cytokine signal” may be confused with “tissue inflammation” as the same level, whereas the former and the latter should be attributed to the cellular level and the organ level, respectively. This leads to ambiguous definitions of the system's intension and extension and its key factors. Therefore, investigation of the mechanisms underlying the formation of mesoscale structures within each level, their functional characteristics, and dynamic changes is crucial to gain deeper insights into the nature of immune‐related diseases. This research can not only reveal the critical role of mesoscale structures in maintaining systematic homeostasis and defending against external pathogens but also provide new insights and strategies for the prevention, diagnosis, and treatment of immune disorders. For example, interactions among immune cells dictate the strength and direction of immune responses in immune diseases. Specifically, the dynamic crosstalk between immune cells plays an important role in the progression of chronic inflammation and autoimmune diseases, providing new avenues for the development of treatment strategies in the immune system.

The human immune system is a vast, intricately layered, and dynamically evolving complex system, and the immune process can be conceptualized as a complex multiscale system involving multiple mesoscale structures within the corresponding level, where the principles of input, output, and boundary conditions are intricately intertwined with immune regulation, encompassing dynamic fluctuations in matter and energy.

For instance, the input process can be envisioned as the ingress of bacteria, viruses, or other antigens into the body, which are subsequently recognized and engulfed by antigen‐presenting cells (APCs) within the immune system [27]. This entry activates the primordial response of the immune system, akin to the infusion of external energy or matter, thereby initiating a cascade of intricate internal transformations. The uptake and processing of antigens are meticulously governed by the MHC polymorphism and the immunoproteasome, resembling the encoding and transformation of input signals, thus laying the groundwork for subsequent immune responses [28]. Furthermore, viral infections can instigate metabolic reprogramming of cells. For example, influenza virus infection may lead to metabolic reprogramming of both immune and epithelial cells, altering their energy metabolic pathways in profound ways [29]. The output can be likened to the immune system's effect on the eradication of external pathogens, including the migration of activated effector T cells and antibody‐secreting cells to sites of infection, such as the respiratory tract, where they eliminate viruses through cytotoxic effects and neutralizing antibodies [30]. This mirrors the discharge of processed matter or energy, with its efficacy directly determining the system's functional performance. Moreover, local inflammatory signals, such as IL‐6 and IFN‐γ, which elicit systemic responses, such as fever, can also be regarded as outputs of the immune system, enhancing the body's defensive capabilities [31]. In this process, metabolic changes in immune cells provide the necessary energy support for the immune response, for instance, effector T cells' transition from oxidative phosphorylation to the utilization of glucose and glutamine to meet their heightened energy demands [32], reflecting intracellular and intercellular energy transformations analogous to energy conversion and metabolic optimization to adapt to internal changes in the corresponding subsystem.

Another example is the interaction between the gut microbiota and the immune system that parallels the boundary conditions in a subsystem, regulating the homeostasis of the immune system [33]. This interaction primarily establishes boundary conditions through various barriers. In a subsystem, boundary conditions serve as interfaces between the system and its external environment, not only constraining the exchange of matter and energy but also preserving the subsystem's homeostasis through dynamic regulation. The physical barrier formed by intestinal epithelial cells resembles the physical boundary, preventing microbial invasion through tight junction protein complexes and mucus layers, akin to a physical boundary restricting the passage of matter [34]. The immune system in the gut detects bacterial components and activates immune responses through pattern recognition receptors, mirroring the signal boundary, which perceives external signals and initiates internal reactions to maintain homeostasis [35]. Metabolites produced by the gut microbiota can regulate the function of immune cells, echoing the metabolic boundary at the organ level, which regulates energy metabolism and material allocation to sustain dynamic system balance [36].

**Figure 3:**
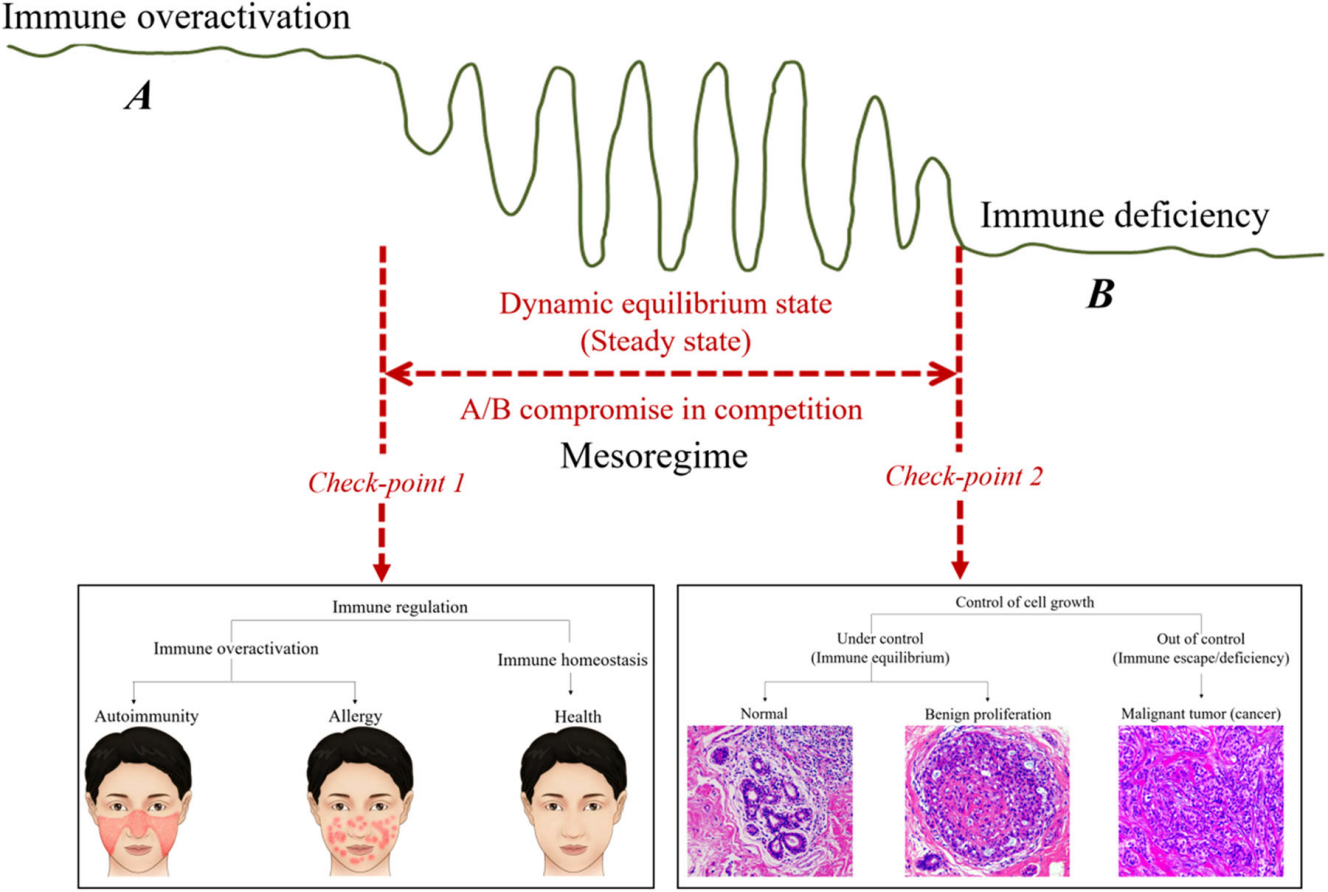
Three characteristic states of the immune system and two critical points. The immune overactivation state and the immune deficiency state are dominated by Mechanism A and Mechanism B, respectively. In contrast, the steady state arises from the compromise in competition between these two mechanisms. Consequently, two checkpoints can be identified as potential regulators in the context of immunological dysregulation.

Incorporation of these concepts into the immune process from the perspective of mesoscience, while considering fluctuations in matter and energy, enables us to attain a more holistic understanding of the complexity and dynamics of the immune system, as well as the intricate interplay among its various components. From the viewpoint of mesoscience, as a result of the interactions arising from these input, output, and boundary conditions, three regimes can exist in the subsystems at each level, and the characteristics and dominant mechanisms at each level are also different. Correspondingly, as shown in Figure 3, three states can be identified, that is, an immune overactivation state (hyper‐immunity or hyper‐inflammation), an immune deficiency state (hypo‐immunity with suppressed immune response), and a dynamic equilibrium state (steady state or immune homeostasis) between these two states.

Normally, when challenged with foreign antigen, specific appropriate responses are initiated that are aimed at restoring homeostasis; however, under particular circumstances, this balance is disrupted, leading to either over‐reactive or under‐reactive immune responses [37]. Over‐reactive immunity suggests too strong immunity that results in autoimmune diseases such as systemic lupus erythematosus (SLE) and allergic diseases such as rashes. Immune overactivation is an important reason for the destruction of the homeostasis of human tissues and organs, which is the reason why most of the pathological changes in viral infections are not caused by viral replication, but by the overactive immune response [38]. Conversely, an underactive immune system leads to immunodeficiency, impairing the body's ability to eliminate malignant cells. This failure in immune surveillance facilitates cancer development, characterized by uncontrolled cellular proliferation, loss of differentiation, tissue invasion, and metastasis [11]. These two opposing pathological states highlight the critical balance required for immune homeostasis, which is in the “normal” immune system that maintains a balance between tolerance to self‐antigens and the ability to respond rapidly to foreign antigens, buffering responses against extremes while maintaining a level of cross reactivity between self‐antigens and non‐self‐antigens to allow rapid responses to a very broad range of antigens [37]. In some diseases, homeostatic control shifts to establish a new state of equilibrium with hypo‐ or hyper‐responses maintained, resulting in chronic immunosuppression or inflammation (Figure 3).

Although both the immune overactivation state and the immune deficiency state involve immunological dysregulation, their therapies should be different due to the fundamentally different mechanisms of immune response. In other words, the three characteristic states and two checkpoints in immune system should not be misinterpreted as two characteristic states and one checkpoint. Therapeutic strategies must be precisely tailored to reestablish the dynamic equilibrium state, either suppressing excessive inflammation or enhancing immune competence, depending on the underlying mechanisms resulting in dysregulation. Understanding these mechanisms is essential for developing targeted interventions in infectious, autoimmune, and oncological diseases. Immune overactivation (e.g., in rheumatoid arthritis) typically requires immunosuppressive therapies, such as cytokine inhibitors, to reduce excessive inflammation. In contrast, immune deficiency (e.g., in primary immunodeficiencies) necessitates immunostimulatory treatments, such as immunoglobulin replacement or cytokine therapy. Thus, while both conditions represent immune dysregulation, their distinct pathophysiological bases demand tailored therapeutic strategies to effectively reestablish immune homeostasis.

As shown in Figure 3, SLE originates from the tolerance breakdown of the immune system toward self‐antigens. This leads to abnormal activation of B cells, which produce large amounts of pathogenic autoantibodies that attack the body's own tissues, triggering inflammation and tissue damage in multiple organs, including the skin. Skin allergy, in contrast, is an abnormal immune response to harmless substances (allergens). Its core mechanism is IgE‐mediated Type I hypersensitivity against innocuous exogenous agents. Although both SLE and allergy involve immune hyperactivation, their pathological mechanisms and clinical manifestations are fundamentally distinct. Understanding these differences is crucial for precision therapy: SLE requires long‐term immunosuppression to control systemic inflammation. Skin allergy management focuses on allergen avoidance and blocking the IgE pathway (e.g., via anti‐IgE biologics). Only by targeting the specific drivers of immune hyperactivation in each disease can we potentially reestablish immune homeostasis.

Deficiency in immune surveillance supports abnormal cellular proliferation. Under physiological immune homeostasis, the mammary ductal epithelium shows minimal or no proliferation. Even when stimulated by factors such as elevated estrogen synthesis, ductal epithelial hyperplasia manifests merely as an increase in normal epithelial cell numbers without significant atypia. In the immune deficiency state, the surveillance capacity of immune cells is compromised. Tumor cells derived from mutated ductal epithelium evade immune detection due to impaired surveillance, facilitating their clonal proliferation and progression to malignant neoplasms (e.g., breast ductal adenocarcinoma) (Figure 3). Elucidation of the regulatory mechanisms governing the transition between immune homeostasis and immunodeficiency is pivotal for tumor early warning systems and preemptive clinical interventions.

From the perspective of mesoscience, the immune overactivation state and the immune deficiency state are dominated by Mechanism A and Mechanism B, respectively, while the steady state is a result of the compromise in competition between these two mechanisms. Both mechanisms A and B are the result of the joint interactions of multiple immunological factors at different levels. Therefore, an in‐depth understanding of the dynamic evolution of the whole immune process at various levels under given external conditions is the key to preventing and treating various diseases related to the immune system. Considering the gut microbiota, for example, the relationship between the host and microbiota has evolved after birth, and each individual will develop a slightly different balance depending on the host, microbes, diet, and drugs. The gut microbiota shapes intestinal immune responses during health and disease, and microbiota‐based modulation could promisingly provide new perspectives for optimizing therapeutic strategies for autoimmune diseases [39, 40].

There is one point that deserves special attention. In the immune deficiency state, for example, cancer, certain therapeutic interventions may alter the dominance of Mechanism B, resulting in a direct transition to the opposite state of immune overactivation. Thus, it is essential to integrate expertise from both oncology and immunology in cancer treatment. A comprehensive evaluation of the patient's tumor characteristics and immune status at all levels could guide the development of individualized treatment strategies and posttreatment medication or health management plans. This approach aims to achieve a new homeostatic balance through compromise in competition between two dominant mechanisms of A and B, that is, the mesoregime.

Even for a single level, it is impossible to gain a complete understanding of the dominant mechanisms in the subsystem and a reasonable explanation of the immune process without identification of the input, output conditions, and environmental factors of the subsystem at this level. For example, in the studies of SARS‐CoV‐2, the structure of the virus and its binding to human receptors including ACE2 were uncovered in an unprecedented short time by combining gene sequencing and protein structure analysis at the molecular level [41, 42], providing decisive data for the design of vaccines and inhibitors [43, 44]. Although SARS‐CoV‐2 causes mild symptoms in most patients, it leads to cytokine storm, tissue damage, and severe acute respiratory syndrome in patients with severe disease. The host response to SARS‐CoV‐2 is complex, and diverse patterns of immune responses have been identified [45]. Recent work suggests that various polymorphisms and differential abundances of ACE2 may help explain some of the different clinical outcomes seen in different patient groups [46]. Distinct immunomodulators have been used to treat SARS‐CoV‐2. However, due to the complexity and diversity of the multilevel and multiscale immune responses in SARS‐CoV‐2, the clinical outcomes are variable. On the other hand, vaccines have been used to prevent the spread of SARS‐CoV‐2. There is an urgent need to explore the pathogenesis of SARS‐CoV‐2 and reveal the complex diversity of immune responses to SARS‐CoV‐2 to seek new treatments and guide vaccine design.

In addition to life science and medicine, an understanding of metabolism, nutrition, and TCM is also very important at the system scale of the corresponding level. Meanwhile, chemistry, physics, engineering, information, and especially complex system science are crucial for understanding dynamic structures at the mesoscale and the evolution within each subsystem at all levels, compromise in competition between different dominant mechanisms, and coupling between multiple subsystems.

### 3.3 Methodological Challenge to Reductionist Approaches

The spatial and temporal dynamic structures of each level and each component in the subsystem at this level also pose a severe challenge to traditional research methods based on reductionism.

During the twentieth century, biological study has been dominated by reductionism, with the idea that complex biological systems can be understood by dismantling them into their constituent pieces and studying each in isolation [47]. However, the organism is a multilevel complex system with spatial and temporal dynamic structures at each level. Traditional research methods based on reductionism cannot map the enormous complexity and diversity of the immune system, and mesoscience has provided a viable alternative.

Considering virus invasion as an example, the significance of multiscale structures, especially mesoscale structures, can be elucidated through system composition and spatiotemporal dynamic structures. First of all, unlike in vitro experiments, the binding of the coronavirus with receptors first takes place at the surface of epithelial cells, mostly the upper respiratory epithelium, and also the digestive tract, resulting in an infection of intestinal epithelial cells [48]. These two different paths will inevitably be affected by the complex cellular fluid environment, and the confined space and heterogeneous environment vary with each individual. Correspondingly, the following questions should be resolved to pave the way for the development of inhibitors. How does the virus reach host receptors? Which cellular fluid is favorable/unfavorable for the survival and transmission of the virus? How can the transmission of the virus to the lower respiratory tract (lung) be stopped? Further, preexisting cross‐reactive immune memory to SARS‐CoV‐2, which largely originates from previous exposure to cold coronaviruses or other pathogens, gives rise to further complexities. Can previous coronavirus infection reserve antibodies cross‐react to recognize SARS‐CoV‐2 [49]? Would these antibodies play protective roles as neutralizing antibodies or pathogenic roles due to promotion of the binding of coronaviruses to the receptors? Answers to these questions require multidisciplinary research on the complexity and diversity of the immune system; correspondingly, the paradigm of traditional scientific research based on reductionism must be shifted [50].

From the mesoscience perspective, when our body is invaded, the normal cellular environment is destroyed. Correspondingly, the spatial and temporal dynamic structures of each level and each component in the subsystem at this level would also change to adapt to the new environmental factors. Meanwhile, the invasive species need to adapt to the new environment gradually. On the other hand, drugs or vaccines should destroy the abnormal environment in which the invaders live to maintain the normal multilevel and multiscale structures of the environment required by human cells and tissues. Therefore, regulation of the dynamic structures at various levels, such as regulation of the internal and external environments of cells, especially the two mechanisms of maintenance and destruction of the normal environment and their interactions, is important for the development of new drugs. The key is how to extract the dominant mechanisms from the multidimensional correlating factors [51].

The regime‐specific behavior of the immunity also indicates some dual functions of the subunits in the immune subsystems [52, 53, 54]. Studies have elucidated that some biomolecules, including RNAs, proteins, and lipid molecules, play opposing roles in tumor regulation depending on the absence or presence of the ligand [55], or subcellular localization [56]. In particular, some investigations have shown that once carcinogenesis has taken place and homeostasis has been disrupted, there is a window of opportunity in which this disruption can be reversed [57]. These mesoregime behaviors provide good evidence for the prediction of the three‐regime feature.

Currently, immunotherapy targeting the immune system is widely used in cancer treatment. Despite success in some patients, most cancer patients still have further tumor growth and metastasis, resulting in the failure of immunotherapy [58]. From the mesoscientific perspective, a possible reason is that the regime shift (e.g., from tumor metastasis to tumor‐immune coexistence, or from tumor‐immune coexistence to tumor elimination) requires precise control of the dominant mechanisms manifested at multiple levels, and there may be two classes of mis‐regulation problems, both of which lead to therapeutic failure. If the regulation is too weak, the critical point of the regime transition cannot be overcome, that is, it still remains in the original state. Conversely, if the regulatory effect is too strong, although it could overcome the critical point, it may trigger an excessive immune response (such as acute inflammation) and lead to severe irreversible damage to the body [59]. In addition, current cancer treatment is usually carried out according to a fixed protocol, without deliberate consideration of the spatiotemporal heterogeneity of the tumor microenvironment and the evolutionary ability of cancer phenotypes to adapt to therapeutic perturbations. The tumor‐immune response often develops dynamically, and treatment often fails due to the emergence of resistant populations [60]. Thus, one possible consideration is to apply evolutionary principles to cancer treatment, shifting the focus from eliminating tumor cells to evolution‐based approaches to inhibit further tumor cell growth and maintain long‐term control [61], that is, remaining in the middle region shown in Figure 3 for a long time. Further research into more effective immunotherapy strategies requires a deeper understanding of tumor‐immune and tumor‐therapeutic interactions at multiple levels in the body, with particular focus on the mesoscale structure in the subsystem at each level. Therefore, revealing the dominant Mechanisms A and B (both of which are composed of many factors) and the two critical points of transition to the A/B dynamic equilibrium state in Figure 3 is the key to disease prevention and therapy.

All these aspects are closely related to the given conditions such as therapeutic protocols, the dominant mechanisms at each level, and the correlations between all these levels. Therefore, to achieve a systematic understanding of the immune system, the overall conditions of the system should be correlated with the dominant mechanisms at each level, and the regulation mechanisms of the dynamic equilibrium structures at all levels should be understood in depth, which urgently requires a shift in the scientific research paradigm and interdisciplinary investigations.

## 4 Summary and Future Perspectives

This paper discusses the multilevel and multiscale complexity of the immune system, with the challenges of immunological research mainly including the following:

1. Mesoscience might provide a promising paradigm to study the complexity and diversity of the immune system, overcome the gaps between multiple levels and scales, and thus enable diagnosis of and development of possible therapies for immune‐related diseases. To achieve this goal, in addition to extensive investigations at the genetic, biomolecular, and cellular levels, greater emphasis should be placed on the mesoscale structures at multiple levels, which is critical for bridging molecular mechanisms with systematic immune responses, and will provide deeper insights into immune regulation, pathogenesis, and development of novel immunotherapeutic strategies. For each level, accurate definition of the input, output, and boundary conditions is a prerequisite for an understanding of the dynamic evolution of the whole immune process.
2. To explore the dominant mechanisms of spatiotemporal structures at each level that govern regime shifts in corresponding subsystems, it is necessary to move beyond traditional reductionism‐based methods and develop multiscale computational frameworks capable of quantitatively characterizing mesoscale structures. By integrating advanced experimental techniques—such as intravital imaging and spatial transcriptomics—with multiscale modeling that explicitly incorporates mesoscale information, and subsequently fusing these multimodal data to infer governing principles or evolutionary laws, critical regime transitions and dominant mechanisms can be effectively revealed. Such an integrated approach not only reconciles reductionist and holistic perspectives but also overcomes the limitations of qualitative methodologies that currently prevail in immunological and biological research.
3. To handle all these challenges and uncover the secrets of the immune system, there is an urgent need to shift the current immunological research paradigm and promote interdisciplinary investigations. Collaborative efforts combining immunology with computational biology, molecular biology, systems biology, and artificial intelligence are urgently needed to decode immune complexity and accelerate therapeutic innovation. In particular, the integration of such diverse fields inherently relies on high‐quality, interoperable data. Therefore, special attention should be paid to the quality of scientific data collected during immunological research, which is critically important for AI applications. While designing an open‐source data/metadata schema for cross‐scale immune data sets, it is essential to strictly adhere to the principles discussed in this paper, as noncompliant data may otherwise lead to significant risks in AI and mechanism analyses.

## Author Contributions

**Ying Ren:** writing – review and editing, investigation, writing – original draft. **Ai‐Guo Wu:** writing – original draft, investigation. **Yu Shi:** investigation, writing – original draft. **Yi‐Fang Ping:** writing – review and editing, investigation. **Jing‐Hai Li:** writing – review and editing, project administration, writing – original draft, supervision. **Xiu‐Wu Bian:** supervision, project administration, writing – review and editing; writing – original draft.

## Ethics Statement

The authors have nothing to report.

## Consent

The authors have nothing to report.

## Conflicts of Interest

The authors declare no conflicts of interest.

## Data Availability

The authors have nothing to report.
